# Supplementary material for: Viable bacterial communities on hospital window components in patient rooms
Source: PeerJ. 2020 Jul 27;8:e9580. doi: 10.7717/peerj.9580 (PMC7391968; doi:10.7717/peerj.9580)
Supplement: Supplemental Information 1 [file peerj-08-9580-s001.docx]

Table S1 DNA sequences for qPCR standards.

| *Clostridioides difficile* TcdA DNA Standard |
| --- |
| CAGGACACACAGTGACTGGTAATATAGATCACTTTTTCTCATCTCCATCTATAAGTTCTCATATTCCTTCATTATCAATTTATTCTGCAATAGGTATAGAAACAGAAAATCTAGATTTTTCAAAAAAAATAATGATGTTACCTAATGCTCCTTCAAGAGTGTTTTGGTGGGAAACTGGAGCAGTTC |
|  |
| Methicillin-Resistant *Staphylococcus aureus* Nuc DNA Standard |
| TTCTTTGACCTTTGTCAAACTCGACTTCAATTTTCTTTGCATTTTCTACCATTTTTTTCGTAAATGCACTTGCTTCAGGGCCATATTTCTCTACACCTTTTTTAGGATGCTTTGTTTCAGGTGTATCAACCAATAATAGTCTAAATGTCATTGGTTGACCTTTGTACATTAATTTAACCGTATCACCATCAATCGCTTTA |
|  |
| *E.coli* 16S rRNA Gene DNA Standard |
| AGATCTGGAGGAATACCGGTGGCGAAGGCGGCCCCCTGGACGAAGACTGACGCTCAGGTGCGAAAGCGTGGGGAGCAAACAGGATTAGATACCCTGGTAGTCCACGCCGTAAACGATGTCGACTTGGAGGTTGTGCCCTTGAGGCGTGGCTTCCGGAGCTAACGCGTTAAGTCGACCGCCTGGGGAGTACGGCCGCAAGGTTAAAACTCAAATGAATTGACGGGGGCCCGCACAAGCGGTGGAGCATGTGGTTTAATTCGATGCAACGCGAAGAACCTTACCTGGTCTTGACATCGTGCCTTCGGGAACGGAGACAGGTGCTGCATGGCTGTCGTCAGCTCGTGTTGTGAAATGTTGGGTTAAGTCCCGCAACGAGCGCAACCCTTATCCTTTGTTGCCAGCGGTCCGGCCGGGAACTCAAAGGAGACTGCCAGTGATAAACTGAGAAGGTGGGGATGACGTCAAGTCATCATGGCCCTTACGACAGGGTACACACGTGCTACAATGGCGCATACAAAGAGAA |
