# Supplementary material for: Viable bacterial communities on hospital window components in patient rooms
Source: PeerJ. 2020 Jul 27;8:e9580. doi: 10.7717/peerj.9580 (PMC7391968; doi:10.7717/peerj.9580)
Supplement: Supplemental Information 2 [file peerj-08-9580-s002.docx]

Table S2. Data summary statistics for bacterial abundance for rooms facing each direction.

|  | **W** | **NW** | **E** | **SE** |
| --- | --- | --- | --- | --- |
| **Mean (log gene copies)** | 3.784357 | 3.18083 | 3.413477 | 3.06874 |
| **Median (log gene copies)** | 3.79421 | 3.250428 | 3.456737 | 3.155334 |
| **SD** | 0.2471845 | 0.4288569 | 0.4555605 | 0.7734787 |
