# Supplementary figures and images for: Viable bacterial communities on hospital window components in patient rooms
Source: PeerJ. 2020 Jul 27;8:e9580. doi: 10.7717/peerj.9580 (PMC7391968; doi:10.7717/peerj.9580)

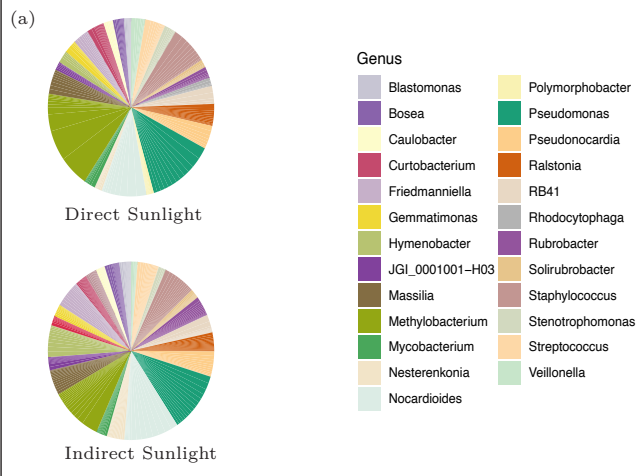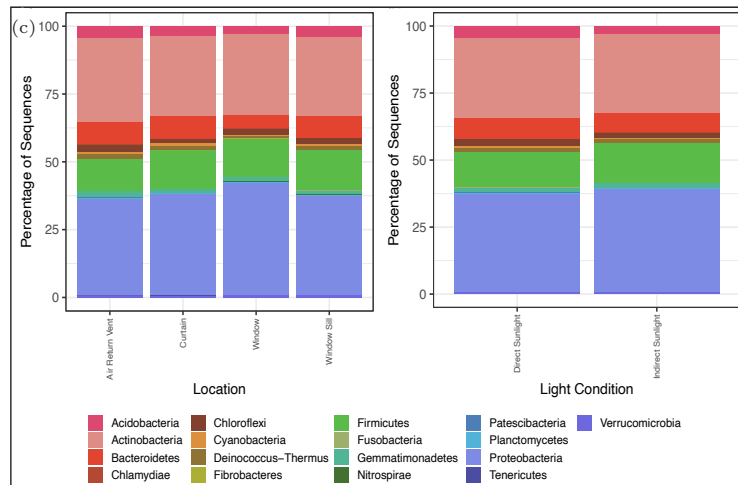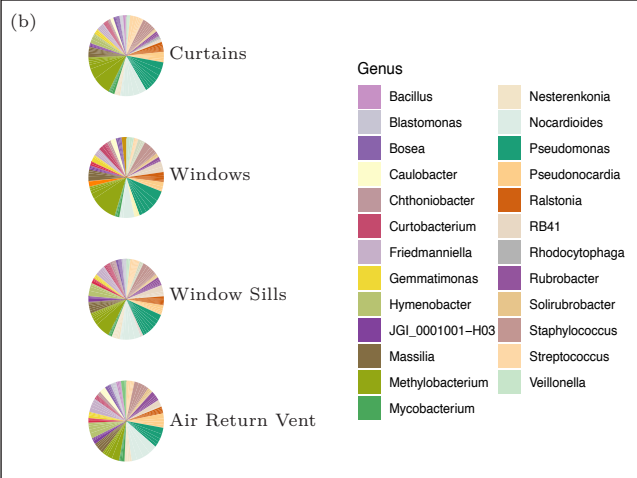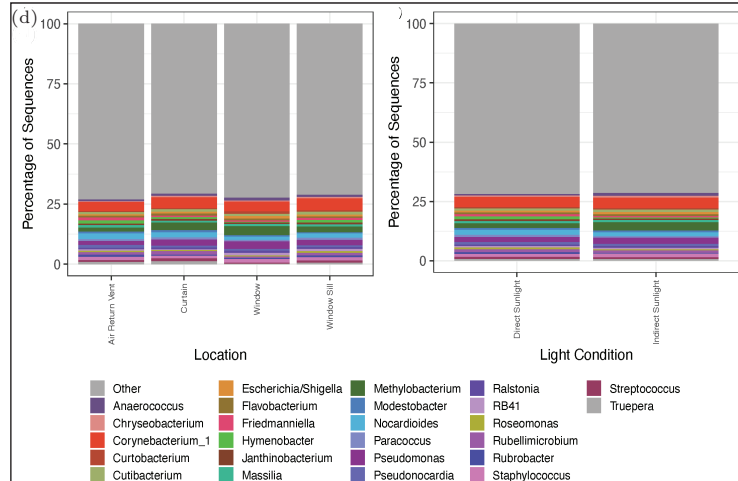

Supplement: Supplemental Information 3 — (A) Pie charts of the 25 most abundant viable bacteria broken down by their Genus in direct sunlight (top) and indirect sunlight (bottom). (B) Pie charts of the 25 most abundant viable bacteria broken down by their Genus on the surface of curtains, windows, window sills, and air return vents respectively (top to bottom). (C) Relative abundance of the identified phyla on each surface tested (left) and in each lighting condition (right). (D) 25 most abundant Genera and their respective relative abundance on each surface tested (left) and in each lighting condition (right). [file peerj-08-9580-s003.pdf]
